# Impacts of Molecular Structure on Nucleic Acid–Protein Interactions

**DOI:** 10.3390/ijms24010407

**Published:** 2022-12-26

**Authors:** Richard P. Bowater, Václav Brázda

**Affiliations:** 1 School of Biological Sciences, University of East Anglia, Norwich Research Park, Norwich NR4 7TJ, UK; 2 Institute of Biophysics of the Czech Academy of Sciences, Královopolská 135, 612 00 Brno, Czech Republic

**Keywords:** cruciform, DNA base sequence, DNA structure, G-quadruplex, nucleic acid–protein interactions, Z-DNA

Interactions between nucleic acids and proteins are some of the most important interactions in biology because they are the cornerstones for fundamental biological processes, such as replication, transcription, and recombination. Nucleic acids can adopt a wide range of structural conformations and this structural flexibility plays critical roles in their interactions with proteins [1]. This Special Issue of the International Journal of Molecular Sciences reports on diverse representatives of such interactions (Figure 1) across a wide range of biological systems.

RNA molecules can adopt great structural diversity due to the range of intramolecular interactions that can be formed within the single-stranded molecules [2]. DNA molecules are typically presented as double-stranded, right-handed B-form helices as their canonical structures, and this maximizes the thermodynamic stability of the molecule [3]. However, a significant body of research emphasizes that alternative, non-canonical DNA structures can exist, including double-stranded, left-handed Z-DNA, but also multi-strand structures such as G-quadruplexes (G4s), intercalated-motifs (i-motifs), triplexes, and cruciform structures. These non-B-DNA structures are usually characterized by the occurrence of single-stranded regions (loops) and/or sites of disrupted base pair stacking (junctions between continuous B-form DNA and the alternative structure) [3].

Variations in the structures of nucleic acids offer different binding sites for proteins, including interactions that focus on a range of sequence- and structure-specific nucleic acid targets. The structures of nucleic acids influence different aspects of biological activity, including physiological and pathological functions [4,5], themes that are addressed in this Special Issue. The collection of articles involve biophysical, biochemical, molecular biological and bioinformatics approaches and cover different biological systems, but there are some common themes among them: several refer to computational biology or bioinformatics approaches, or highlight additional information in DNA sequences [6,7,8,9]; several articles refer to different types of non-B DNA structures [10,11,12], with specific interests in quadruplexes [13,14,15,16]; several articles address different approaches and outcomes from proteins binding to DNA structures [11,12,13,17].

The wealth of DNA sequence information provided by genome-sequencing projects has brought new insights into the primary sequences of genomes and also about possible sequence-dependent local secondary structures [3,18]. Advances provided by such genome sequences are exemplified by the Human Genome Project, with complete telomere-to-telomere sequences being finalised in 2022 [19,20]. As highlighted, this Special Issue includes several articles that report on computational biology or bioinformatics studies of DNA sequences [6,7,8,9], identifying unexpected and additional information within them (Figure 1A). In a mini review, Bartas et al. summarize current knowledge about the amino acid composition of various nucleic-acid-binding proteins, highlighting differences across proteins that bind in a sequence-specific manner compared to those that recognize local non-B-DNA structures and those that recognize both types of properties of nucleic acids [6]. Bioinformatic studies of repetitive DNA sequences in *Drosophila melanogaster* polytene chromosomes show that chromatin structure plays a crucial role in the regulation of gene activity [7]. Recent advances reported by Choi et al. demonstrate that nucleic acids may provide useful tools for building complex logic circuits [8]. Finally, for this grouping of articles, Víglaský explores the organization of genetic information in nucleic acids using a novel orthogonal representation, which proves to be useful in predicting the likelihood of particular regions of nucleic acids to form non-canonical motifs [9].

From the earliest days of genome sequence analysis, it was recognized that natural DNA molecules contain a wide array of repeating sequences [3]. These types of sequences are particularly prone to adoption of non-canonical DNA structures, such as G4s, triplexes, and cruciforms (Figure 1B), which are all explored in this Special Issue [10,11,12,13,14,15,16]. Zhao and Usdin review the range of structures that can form in specific trinucleotide repeats, highlighting how their expansion in length is important in the pathology of fragile X-related disorders in humans [10]. Left-handed Z-helices can form in both DNAs and RNAs with appropriate sequences, and searching databases containing protein structures identified novel proteins predicted to bind them [11]. A different type of repetitive DNA sequence, inverted repeats, can adopt cruciform structures and many proteins have now been validated to bind to them [12]. A series of articles provide insights about G4s. Bezzi et al. suggest that putative G4s found in the SARS-CoV-2 RNA genome and the cellular proteins likely to interact with them may constitute interesting targets for antiviral drugs [13]. Putative G4s in viruses are explored further in a study that reveals a positive correlation between their frequencies in double-stranded DNA viruses and their hosts from archaea, bacteria, and eukaryotes, indicating that their close coevolution leads to reciprocal mimicking of genome organization [14]. The potential of compounds to target G4s was explored for Rhodamine 6G, which was shown to have high selectivity for G4s with parallel topology [15]. A bioinformatic study combined with circular dichroism measurements identified a stable G4 that is evolutionarily conserved amongst plants sensu lato (in Archaeplastida), and this may form an additional layer of regulatory networks [16].

The wide array of structures that can be adopted by nucleic acids offer different opportunities for proteins (and other molecules) to bind to, leading to different types of outcomes [4,5]. Some proteins recognise sequence-specific targets, but an increasing number are being shown to interact with non-canonical structural aspects of nucleic acids (Figure 1C). We have already referred to some articles in this Special Issue that describe such interactions [11,12,13]. Another study took advantage of available datasets and discovered new correlations between specific amino acid deviations in p53 proteins, showing a direct association between specific amino acid residues in the protein and changes in p53 functionality, and further highlighting the importance of p53 protein in processes that influence lifespan and aging [17].

To summarize, this Special Issue of the International Journal of Molecular Sciences reports on representatives of interactions between nucleic acids and proteins, with an emphasis on understanding how the structure of the nucleic acid influences such interactions. It is important to characterize these molecular complexes because many are essential requirements for the viability of cellular life due to their involvement in fundamental aspects of nucleic acid metabolism. It is now clear that the structural flexibility of the nucleic acids plays critical roles in their interactions with proteins, with important implications across a range of human diseases, including cancer and some infectious diseases. A deeper understanding of these molecular interactions will require the use of complementary methods and techniques [1]. As is described in this Special Issue, biophysical, biochemical, molecular biological and bioinformatics approaches will deliver useful advances across a wide range of biological systems.

## Figures and Tables

**Figure 1:**
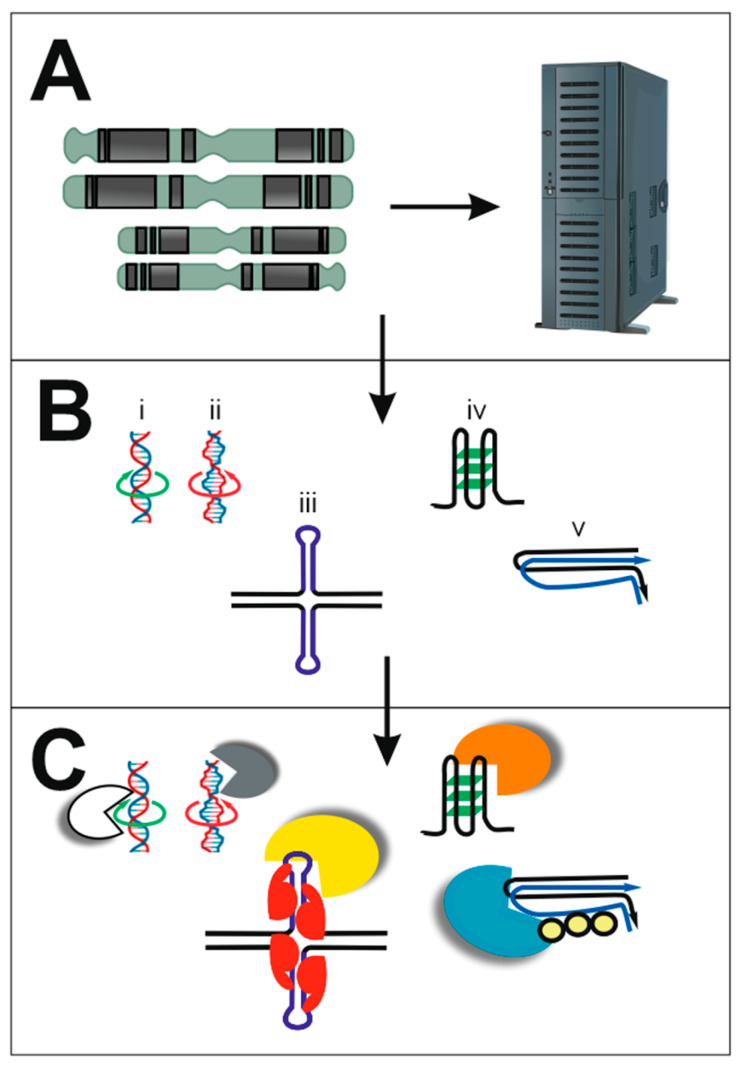
Nucleic acids provide a wide array of sequences and structures that are useful for biological processes. (**A**) Genome sequences store large amounts of information that can be accessed for biological processes and structure prediction by various computational algorithms or for building complex logic circuits. (**B**) Nucleic acids can adopt a range of structures, including those indicated. From left to right: (i) double-stranded, right-handed B-DNA; (ii) double-stranded, left-handed Z-DNA; (iii) two intramolecular hairpins can come together to form a cruciform; (iv) G-quadruplex, formed from four strands that can be parts of one molecule (as shown) or from different molecules; (v) a triplex can be formed when three strands come together, which can be parts of one molecule (as shown) or from different molecules. (**C**) The variety of structures of nucleic acids offer opportunities to be recognised by other molecules, such as proteins. The range of structures shown here may be recognised by different proteins, as indicated by the different colours.

## Data Availability

No new data were created or analyzed in this study. Data sharing is not applicable to this article.
